# Tea in the Morning and Khat Afternoon: Health Threats Due to Khat Chewing

**DOI:** 10.7759/cureus.12363

**Published:** 2020-12-29

**Authors:** Iana Malasevskaia, Ahmed A Al-Awadhi, Lubna Mohammed

**Affiliations:** 1 Obstetrics and Gynecology, Obstetrics/Gynecology Private Clinic, Sana'a, YEM; 2 Obstetrics and Gynecology, California Institute of Behavioral Neurosciences & Psychology, Fairfield, USA; 3 Anaesthesiology, German-Yemeni Hospital, Sana'a, YEM; 4 Internal Medicine, California Institute of Behavioral Neurosciences & Psychology, Fairfield, USA

**Keywords:** cathine, khat, khat and liver disease, khat and health impact, effects on general health, chewing khat, amphetamine-like plant, cathinone, catha edulis, health hazards

## Abstract

Khat or qat (Catha edulis) is a flowering plant with an Arabic name قات, regularly named as qāt, also is known by various descriptive names, such as Abyssinian tea, Somali tea, Arabian tea, Miraa, Jima, and Kafta in its endemic regions of the Horn of Africa and the Arabian Peninsula. Fresh leaves and tops of khat are chewed or dried and consumed as a tea to attain a state of euphoria and excitement; it also has appetite-reducing effects. Traditionally, khat is used as a socializing habit in Yemen and is also widely cultivated because of its high income. However, in recent years the plant has been reported in England, Wales, Rome, Amsterdam, Canada, Israel, Australia, New Zealand, and the United States. Although it is believed that khat is a relatively low-risk drug, it's associated with an increased risk for various medical complications, including dental and oropharyngeal disease, gastrointestinal, cardiovascular, neuropsychiatric, obstetric, and even can be the cause of cancer. Our goal in this review article is to revise and determine the relationship between chewing khat and its health issues. Additionally, we tried to determine the mechanism involved in health hazards due to consuming the khat.

## Introduction and background

Approximately 20 million people worldwide put their health at risk using a daily habit of khat-chewing, some for pleasure and some as cultural significance [1]. The earliest reference to khat (Catha edulis Forsk), which was imported from Turkistan, appears around 973-1053 AD by Al-Biurni, who carefully compiled information about a new drug called khat, which was used to cool the stomach and liver and relieve the biliousness. Additionally, some reports indicate that reference to khat may be as early as 1332 AD in an Arabic manuscript preserved in Paris in the Bibliotheque National. In the 13th century, physicians prescribed khat to reduce the fatigue of soldiers. As a recreational habit, khat chewing may have begun in the Southern Red Sea region before the 14th century [2]. The earliest description of khat in western literature was in 1697 by the French Barthélémyd' Herbelot de Molainville when he traveled to Yemen; he specifically said (translated) "It is made from a seed which is unknown to us, but which has been forbidden by doctors of the law in Yemen, because it is too strong, and affects our brain". However, in 1975 the Laboratories of the United Nations first discovered that cathinone was the biochemically-active ingredient of khat [3].

Khat, also known as Qat, Kat, Chat, Miraa, and Quaadka, is a flowering plant natively found in the Arabian Peninsula to the Horn of Africa [4]. Khat (Catha edulis) is a leaf cultivated in Kenya, Yemen, Ethiopia, and Somalia [5]. Leaves of fresh khat contain the alkaloids of the phenylpropanolamine type, which contains two psychoactive stimulants cathinone cathine (S, S-(+)-norpseudoephedrine) and (S- (-)-α-aminopropiophenone). The psychoactive substances in khat act on two neurochemical pathways - noradrenalin and dopamine [6-8]. Other phenylalkylamine alkaloids were found in khat leaves are the merucathinone, phenylpentenylamines, and merucathine pseudomerucathine, which probably contribute less to the stimulant effects of khat [7, 8].

Khat acts as a euphoriant, having amphetamine-like characteristics [8]. It has been proposed that cathinone releases serotonin in the central nervous system (CNS) like amphetamine, and both of those substances stimulate the release of dopamine from CNS dopamine terminals, thereby increasing dopaminergic pathways [6, 8]. Cathinone is a much powerful stimulant than cathine and is generally believed as the most important component. Also, cathinone is unstable in the presence of oxygen and decomposes within a few days of harvesting or, if dried, oxidizing at room temperature [6]. Khat contains more than forty alkaloids, tannins, glycosides, vitamins, amino acids, and minerals [7]. The euphoric effect begins shortly after the chewing starts, suggesting that absorption starts from the oral mucosa. The maximum effect starts after 15-30 minutes, and the metabolism of cathinone is rapid, occurring mainly during the first passage through the liver. Most cathinone is metabolized to norephedrine and is excreted in this form. Cathine has a serum half-life in humans of about three hours and is slower in action. When taking khat, large amounts of non-alcoholic drinks are consumed; therefore, there is a pharmacological synergism with drinks containing methylxanthines (e.g., tea and cola), enhancing the effects of khat [8]. 

**Figure 1:**
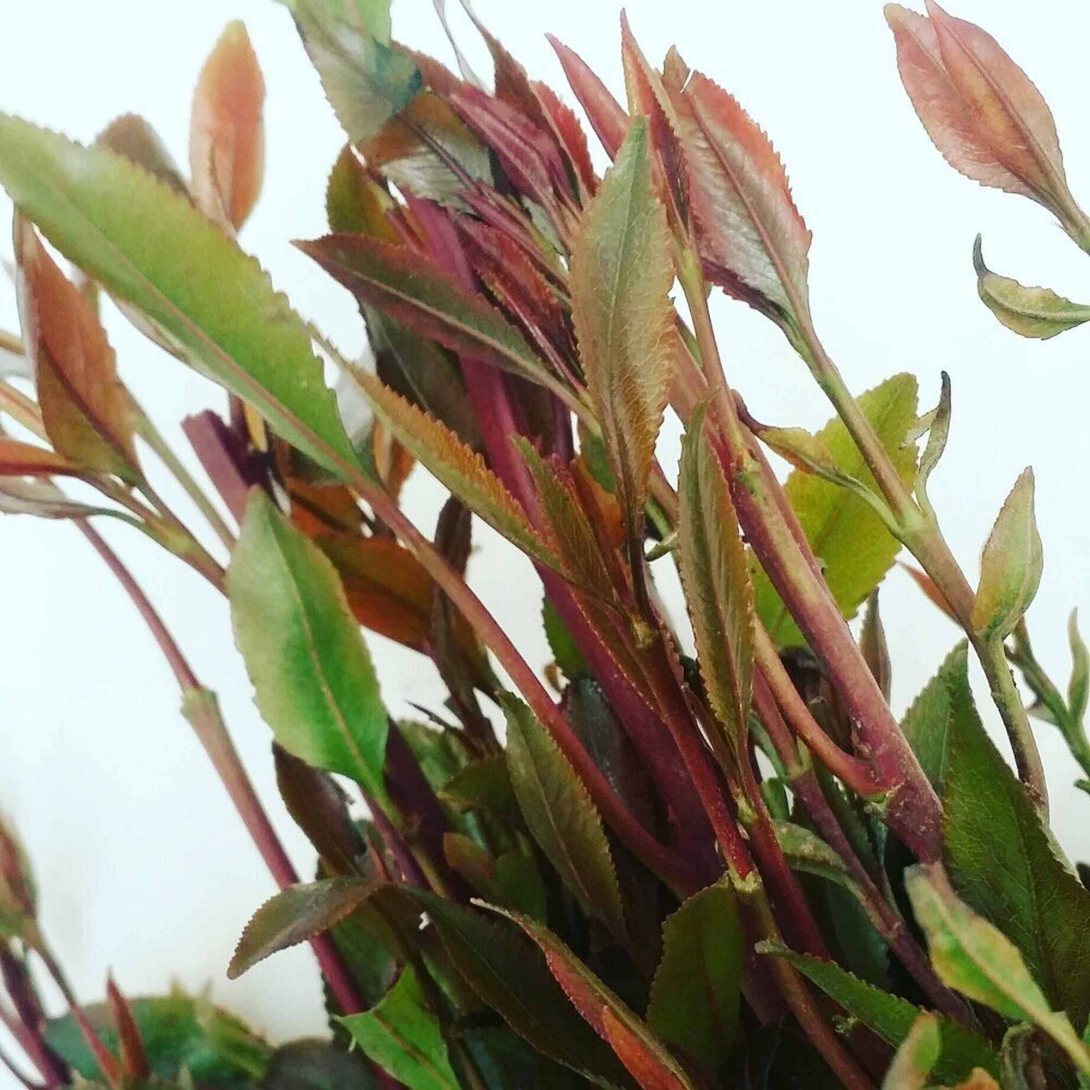
Fresh branches of khat (Catha edulis) plant Image credits to Malasevskaia I.

The climate and environmental conditions determine the chemical profile of khat leaves. In the Yemen Arab Republic, about 44 different khat types originate from foreign countries' geographic areas [7]. Fresh branches of the khat plant are demonstrated in Figure 1.

As early as 1935, international organizations were confronted with the problems associated with khat chewing [2]. In 1965, the World Health Organization (WHO) noted khat's abuse as a regional problem that should be controlled in Somalia, Ethiopia, Kenya, Saudi Arabia, and Yemen. Accordingly, khat was not classified under the Single Convention on Narcotic Drugs. Khat chewing is not lawfully legalized in most countries, such as Yemen, Ethiopia, Somalia, Djibouti, and Kenya, where the habit is performed. At the same time, it is illegal in Saudi Arabia, Malaysia, and the USA [9]. In 1980, the WHO categorized khat as a substance of abuse that can cause mild to moderate psychological dependence [9]. 

The exact number of people worldwide who use khat is unknown; however, it is estimated to be from about 10 million, predominately in Yemen, Somalia, and Ethiopia, and is widely used in Yemen, even by children. The higher prevalence in males than in females [3]. Studies have estimated prevalence to be 50% for females and 80% for males in the capital of Yemen, Sana'a, at age fifteen and above. Also, between 15-20% of children under age 12 are khat's daily consumers [3, 10]. People of those countries usually spend most of the day buying and chewing khat, severely affecting working hours and the national income in general, and the family and society in particular [3].

Several studies have disclosed that khat's regular consumption seriously affects the user's social and economic condition [11]. Khat's daily cost may also affect household income to satisfy nutritious food, education, home improvement, or other family needs, leading to financial problems and family breakdown [11]. Additionally, this causes absence from work, absenteeism from class and low academic performance of the students, and unemployment [9]. Lastly, the death rate is remarkably higher among khat chewers due to chronic illnesses such as heart disease and stroke compared with non-khat-chewers [11]. 

The current study's main aim was to analyze khat's effect on organ systems and the mechanism which is involved in health hazards due to regular consumption of khat.

## Review

Methods 

An extensive literature search on PubMed and non-PubMed indexed journals was performed. Literature databases were searched to identify the information available about the khat plant and its effects on general health, using the following terms: "khat", "khat and health hazard", "the effects of khat", "Catha Edulis". The full text of the articles eligible for our study was reviewed and included in the review. We included only the articles written in English related to khat and health hazards on humans and animals. Only the most relevant research articles were included.

 Acute Effects of Khat Chewing 

Khat chewers state their subjective experiences of khat use positively when consuming small amounts [8]. They describe a feeling of well-being, joy, excitement, a sense of euphoria, increased energy levels, feelings of increased mental alertness and arousal, talkativeness, increased ability to concentrate, mydriasis, and blurred vision; along with increased blood pressure, hyperthermia, increased heart rate, increased locomotor activity, suppression of appetite, improvement in self-esteem, and an increase in libido [1, 3, 8, 12, 13]. Some people describe increased imagination and ideas [1]. Also experienced are an enhanced imaginative ability and capacity to associate ideas, a subjective improvement in work performance, and improved communication ability [8]. 

Khat Withdrawal Symptoms 

After chewing, unpleasant after-effects tend to dominate the experience: insomnia, loss of appetite, numbness, lack of concentration, and low mood [8, 12, 13]. Some chewers also experience unpleasant effects during the chewing process, describing anxiety, irritability, craving, tension, a slight tremor, restlessness, and hypnagogic hallucinations [8, 12-14]. 

Among people who reported experiencing withdrawal symptoms, 66.3% said that the withdrawal symptoms caused problems in their lives, such as their jobs or relationships. The biggest concern with khat withdrawal is low mood and depression, which increase the chance of relapsing or turning to other substances to fill the void [14]. 

Signs of Use

**Figure 2:**
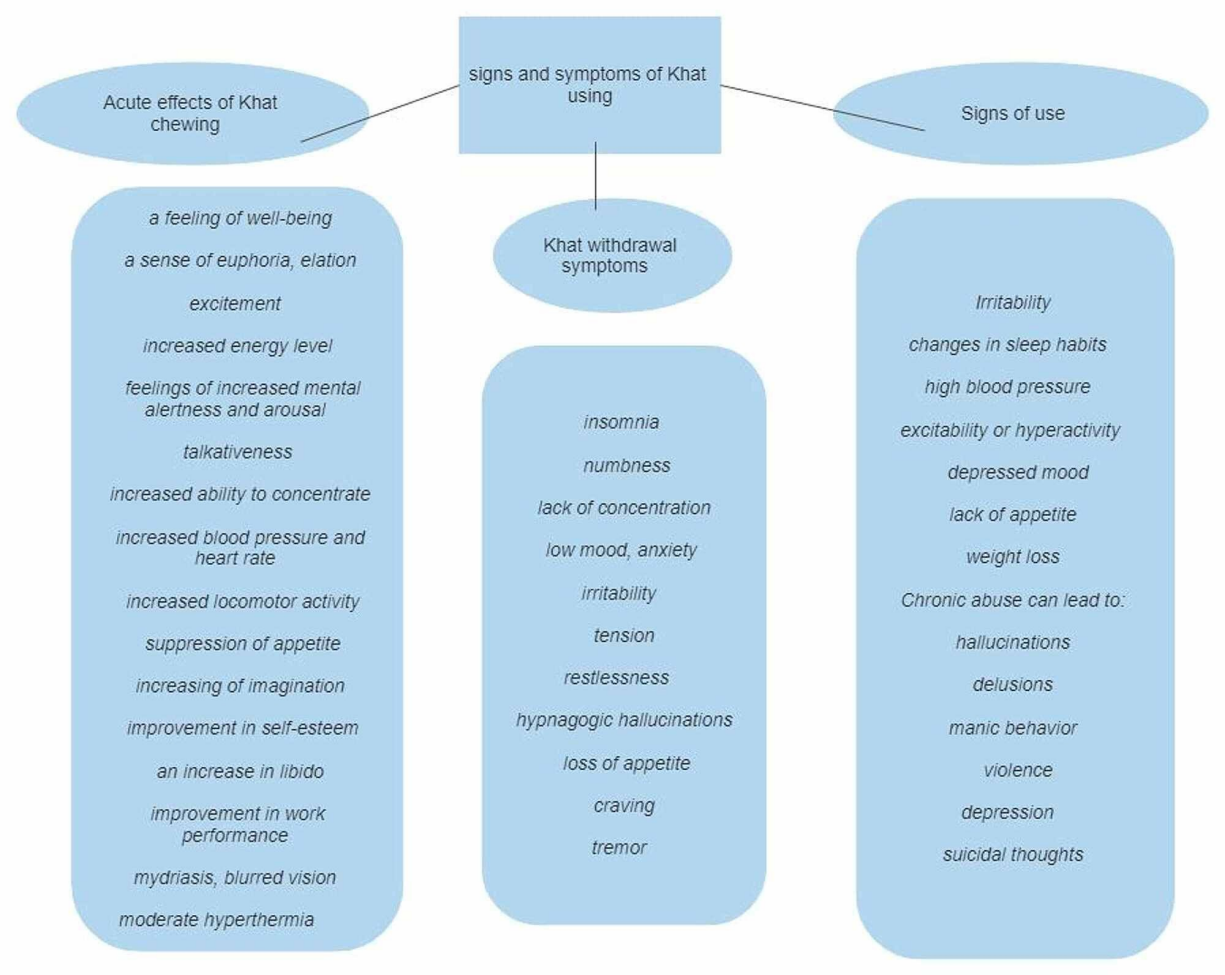
A summary of acute and chronic signs and symptoms of khat using Image created by Malasevskaia I.

There are some behavior and physical signs and symptoms that someone who is using khat can show. The users can express irritability, changes in sleep habits, high blood pressure, excitability or hyperactivity, depressed mood, lack of appetite, weight loss [1]. However, as seen in Figure 2, chronic abuse can lead to adverse health effects such as hallucinations, delusions, manic behavior, violence, depression, suicidal thoughts [14].

Health Threats of Using Khat 

Chronic khat use results in increased risk for several diseases such as cardiovascular, central nervous system, gastrointestinal, genitourinary, obstetric, and other diseases. 

*Khat and Central Nervous System * 

The main active ingredient of khat is cathinone, which is an amphetamine-like substance. Khat chewers report feeling of wellbeing, increased energy levels, a sense of euphoria and excitement, increased alertness, improvement in self-esteem, increased ability to concentrate, an increase in libido, enhanced imaginative ability, improvement in the ability to communicate, the capacity to associate ideas, and subjective improvement in work performance when consumed in small amounts. Minor reactions include over-talkativeness, over-activity, insomnia, irritability, anxiety, agitation, and aggression. However, chewers can show a range of experiences, from minor reactions to developing a psychotic illness [15]. Though intoxication with khat is self-limiting, chronic consumption can lead to mental health impairment, possibly contributing to personality disorders and mental deterioration [2]. The main psychiatric manifestations linked to the use of khat are a short-lived schizophreniform psychotic illness, mania, and, more rarely, depression [15]. 

There are two main psychoses outlined in the literature related to khat consumption: a manic psychosis and paranoid or schizophreniform psychosis (similar to an amphetamine-like psychosis). 

Schizophreniform psychosis*:* Recent increase in khat use or a moderate consumption results in fear, paranoid delusions, ideas of reference hostile perception of the environment, auditory hallucinations (frequently of a persecutory or threatening type), thought alienation, and a tendency to isolate themselves or to display aggressive behavior towards others. If khat consumption is stopped at this time, symptoms' resolution usually occurs within a short period of time (3-11 days); however, there is a predisposition for the psychosis to recur if khat chewing is restarted [8]. 

Manic psychosis:* *Several authors have described a manic-type psychosis. The first case in the USA was reported in 1982 [8]. The patient presented with hyperactivity, shouting, speech pressure, grandiose delusions with the flight of ideas, and an unstable mood varying from euphoria to anger. The patient had consumed khat for the first time, chewing about 24 leaves (this is equivalent to a single dose of khat), and symptoms subsided spontaneously within about eight hours of chewing [8]. 

Confusion states are rare; usually, the paranoid reactions occur in clear consciousness, confusion, and disorientation as a transient phenomenon in the khat user may occur, even without psychosis. The level of sympathetic arousal is stronger in acute khat intoxication than in heavy prolonged use, where some sympathetic tolerance occurs; this might help distinguish between these situations [8].

Several studies show that there is a relationship between khat chewing and psychosis. A cross-sectional and case-control study was done in Somalia by Odenwald M et al. Trained local interviewers screened 4,854 randomly selected persons from among the general population in Hargeisa, North-West Somalia, for disability due to severe mental problems. The identified cases were interrogated based on a structured interview and compared to healthy matched controls. Psychotic symptoms were assessed using the World Health Organization (WHO) items, quantified with the Positive and Negative Symptoms Scale and Composite International Diagnostic Interview. Statistical testing included the Student's t-test and analysis of variance (ANOVA). Local interviewers established that rates of severe disability due to mental disorders were 8.4% among males (above the age of 12). The clinical interview validated that in 83% of positive screening cases, psychotic symptoms were the most prominent manifestations of psychiatric illness. Roughly, cases with psychotic symptoms had started to consume khat earlier in life than matched controls and had been using khat for 8.6 years before positive symptoms emerged. In contrast to controls of the same age, in most cases with psychotic symptoms, a binge use pattern (> two 'bundles' per day) preceded the onset of psychotic symptoms [16]. 

Another case-report done presented by Tesfaye E et al. in Ethiopia demonstrates the relationship between khat consumption and khat-induced psychotic disorder. A 33-year-old male started using khat in a small amount in 2004 for recreational purposes; however, since 2016, his khat consumption and amount of time using it increased, which caused frequent absence from work, and conflict with his boss. Besides, his khat use brought him into frequent conflicts with his wife and divorce in late 2016. Later he began to have visual hallucinations and hearing voices; furthermore, he also reported that sometimes while chewing khat, he felt detached from himself and observed himself from outside. In 2018, his illness worsened to the extent he attempted to kill himself using a rope in the absence of a depressed mood. After he agreed to quit khat use, his illness improved in two weeks of his sobriety from khat consumption [17]. This case demonstrates that the use of excess khat above two bundles for a prolonged duration can manifest with psychotic episodes. 

Depression, anxiety, and stress*:* A cross-sectional study was conducted on 642 students from Jazan University, Kingdom of Saudi Arabia, by Al Bahhawi T et al. Khat chewing habit is prevalent among the Jazan population in Saudi Arabia. They used multistage sampling, a 21-items Depression, Anxiety, and Stress Scale questionnaire to collect the data, and analyzed it using SPSS software (IBM Inc., Armonk, USA). Results revealed that moderate depression was prevalent among 53.6% of the sample; anxiety was found among 65.7%, while 34.3% suffered from stress. Strong association with higher mean scores for symptoms of depression, stress, and anxiety with p-values <0.05 was among female students. Moreover, khat chewing and excess caffeine consumption among students were associated with higher rates of anxiety symptoms. Khat use was significantly associated with higher mean scores of depression and anxiety among females and a higher mean score of anxiety among males [18].

Khat and Cardiovascular System

Cathinone, which is found in khat, has direct effects on the cardiovascular system due to indirect sympathomimetic activity increasing heart rate and blood pressure in humans, significantly increasing systolic and diastolic blood pressures, which persist for three to four hours after the onset of khat chewing [15, 19]. Khat chewers experience an increase in heart rate, body temperature, and sweating. This is associated with cold extremities, a clinical manifestation of peripheral vasoconstriction [19]. 

Hypertension*:* Based on a comparative study by Getahun W et al., done in Butajira, Ethiopia, chewing khat regularly is associated with elevated mean diastolic blood pressure, consistent with the peripheral vasoconstrictor effect of cathinone. Systolic and diastolic blood pressure were compared among adults aged 35-65 who reported chewing khat during the last five years to those who never chewed khat during the same period. The comparative groups, non-chewers (n=330) and chewers (n=334), were identified from among the general population through a house-to-house visit using a screening questionnaire. The prevalence of hypertension was higher among khat chewers (13.4%) than non-chewers (10.7%), and a considerably high percentage of chewers (29.9%) than non-chewers (20.6%) had sub-optimal diastolic blood pressure (> 80 mmHg). The mean diastolic blood pressure was higher among khat chewers than non-chewers. Similarly, khat chewers had a significantly higher mean heart rate than non-chewers. There was no significant difference in mean systolic blood pressure between the two groups [20]. 

Additionally, another comparative cross-sectional study was undertaken from October 5, 2018, to February 15, 2019, in the Gurage zone, southern Ethiopia, by Geta T et al. A total of 1,200 adults (600 non-chewers and 600 chewers) aged 18-65 years were chosen using a convenience sampling method; the data was collected by an interviewer-administered questionnaire plus blood pressure measurements and were carried out at a fixed time of the day in the morning (7 am -10 am). The mean values of the systolic blood pressure along with diastolic blood pressure were higher in chewers than in non-chewers (p<0.001), and the prevalence of diastolic blood pressure (>80mmHg) was significantly higher among khat chewers than in non-chewers (17.4% versus 8.7%, p < 0.001) [21]. 

Coronary syndrome and myocardial infarction*: *A descriptive, cross-sectional study was done by Al-Motarreb A et al. in Yemen. Patients referred for coronary angiogram were enrolled for a six-month time from November 15, 2008, to May 15, 2009. Doctors interviewed all patients, using a predesigned questionnaire, interviewing the patients just before the catheterization procedure. The study procedures were explained, and consents were obtained and approved. Data were collected from patients presented with acute myocardial infarction (AMI) and underwent coronary angiographic study in the cardiac center, Al-Thawrah Hospital, Yemen. Patients were divided into three groups: group 1 (diabetic and khat chewers), group 2 (khat chewers and non-diabetic), and group 3 (diabetic and non-khat chewers). Data, including demographics, clinical presentations, and coronary angiographic findings, were analyzed and compared among the three groups. The result shows that out of 347 AMI patients, 55 (16%) were both khat chewers and diabetic (group 1), 219 (63%) were khat chewers and non-diabetic (group 2), and 73 (21%) were diabetic and non-khat chewers (group 3). The male-to-female ratio was nearly 3:1. Furthermore, khat chewers were more likely to be smokers and less likely to have hypertension than non-chewers. All patients participating in the study underwent diagnostic coronary angiography during the admission. Patients from group 1 were more likely to have left anterior descendent (LAD) artery total occlusion, first obtuse marginal (OM1) remarkable lesions (>70% occlusion), and severe left circumflex (Lcx) artery lesions when compared to other groups. Group 2 (khat chewers only) patients had non-significant lesions (58%), as well as normal coronary arteries (44%) comparing to the other two groups who were diabetic as well. In contrast to group 1 and 2 patients, group 3 patients had two to three vessels affected, showing significant LAD coronary artery occlusion (70-95% occlusion), and Lcx occlusion, total right coronary artery (RCA), and RCA stenosis (>70% occlusion) [22]. 

Khat contains cathinone, a natural amphetamine, which induces coronary spasm, which induces coronary artery spasm and precipitates AMI. In addition to this theory, cathinone produces myocardial oxygen demand-supply mismatch, through increased heart rate and blood pressure, especially in the first few hours after khat parties [22]. 

Stroke*:* A case report by Kulkarni SV et al. shows that khat chewers can also develop a cerebrovascular stroke. A 47-year-old man from Yemen, Sana'a, presented with sudden onset left-sided weakness with a deviation of mouth to the right side. There was no comorbidity history like type 2 diabetes, hypertension, or ischemic heart disease. As well as no history of alcohol, drug intake, or smoking. The patient was a regular khat chewer for the last 30 years (five to six packets a day-each packet containing nearly 100 g spreading over six to eight hours over the day), and there was no significant family history. Standard investigations such as complete blood count (CBC), erythrocyte sedimentation rate (ESR), random blood sugar (RBS), serum electrolytes, and urine analysis were normal. Electrocardiography and transthoracic echocardiography, carotid, and vertebral artery Doppler studies were also normal. Additionally, the magnetic resonance angiogram of vertebral, carotid, and cerebral arteries was normal. A detailed workup for common etiologies of stroke in the young people included lipid profile, anticardiolipin antibodies, protein-S, serum homocysteine level, antiphospholipid antibody normal, and tests for infections (hepatitis B and C, human immunodeficiency virus) and syphilis were negative. Computed tomography of brain (plain and contrast) showed acute right middle cerebral artery infarction. A diagnosis of left hemiplegia due to right middle cerebral artery infarction was established and treated accordingly; additionally, the patient was advised to discontinue khat chewing [23]. 

Another case report presented by Attafi MI et al. in Jazan, Saudi Arabia, described a 35-year-old male patient who was admitted to the hospital with initial symptoms of left limb weakness and loss of consciousness. He was diagnosed with a hemorrhagic stroke with hypertension and started on symptomatic and supportive treatment. Lab analysis of serum biochemistry showed normal renal and liver function tests. His symptoms were gradually improved, and upon reviewing the previous history, this patient had never been diagnosed with chronic diseases, but he was chewing khat daily. His general toxicological screening results were negative for amphetamine, cocaine, opiate, barbiturate, and tricycle antidepressant in the blood sample and positive for the amphetamine-like substance in the urine sample. On further investigation, the confirmatory analysis by liquid chromatography-tandem mass spectrometry (LC-MS/MS) in urine was detected and quantified cathine and cathinone. The serum concentration of cathine was more than a hundred times that of cathinone, indicating that symptoms are most likely due to cathine. The patient was treated, and his condition improved following the cessation of khat using [24]. 

Arrhythmia: Khat chewing can cause palpitations, chest discomfort, and even sudden death. To study khat chewing's cardiac rhythm effects, Jayed D et al. selected 60 khat-chewing Yemeni individuals. They divided them into two groups: 30 were cardiac patients, and 30 were non-cardiac individuals. All 60 individuals underwent 24-hours Holter monitoring for two consecutive days; a khat-free day followed by a khat-chewing day. The two groups were matched according to sex, age, smoking habit, systolic, and diastolic blood pressure. Non sustained ventricular tachycardia (VT) was defined as three or more wide QRS complexes at a rate of 120 beats/min and for a period of fewer than 20 seconds. The non-sustained VT was found in seven (23.3%) of the 30 cardiac patients on a khat-chewing day compared to two patients (6.6%) on a khat-free day (p<0.01). A significant difference was also seen among the normal individuals; one patient (3.3%) developed VT's short runs on a khat-chewing day than non-VT on a khat-free day. Serious arrhythmias occur in cardiac and non-cardiac individuals during khat chewing days, although they are more prominent among cardiac patients [25]. However, further studies are needed regarding khat’s effects on cardiac rhythm. 

*Khat and Gastrointestinal System* 

Mouth disease*:* Based on a cross-sectional study conducted by Astatkie A et al. on a sample of 1,255 university students in southern Ethiopia, khat chewing is associated with many mouth health problems. The long-term regular khat chewers more frequently reported the oral symptoms including pain in the jaw joint while chewing (16.8%), mouth ulcers (13.6%), toothache (16.6%), dryness of the mouth (14.4%), and a burning sensation in the tongue or other parts of the mouth (13.0%) [26]. 

Al-Kholani AI performed another cross-sectional study on 730 patients visiting the government dental college's outpatient dental clinics, University of Sana'a, Yemen. The patients were divided into two groups: khat chewers (n=336) and non-chewers (n=394) for comparison purposes. Only patients who had a history of chewing khat for more than three years, not less than four hours a day, and not less than four days per week were considered. The data show that quite a large number of khat chewers (88%) and non-chewers (91%) used paste regularly for maintenance of oral hygiene; however, 9% of chewers did not use toothpaste, as compared to 5% of non-chewers. About 49% of non-chewers had good oral hygiene status compared to only about 15% of khat chewers. The incidence of gingival bleeding was higher in khat-chewers than in non-chewers, and (58%) of chewers had halitosis. Around 23% of chewers complained of difficulty in mouth opening compared with only about 1% of non-chewers [26]. 

Multiple gastrointestinal disorders*: *A cross-sectional study was conducted in January 2010 on 1,005 Ambo University students in Ethiopia by Nigussie T et al. Seven hundred and twenty (71.6 %) participants were males, and 994 (98.9%) were in the age group of 15-24 years. The prevalence of gastritis was in 580 (57.7%); hemorrhoids in 54 (5.4%) and that of dental problems (carries, decay, filling); constipation in 235 (23.4%) and extraction was in 225 (22.4%) of all study participants. Gastrointestinal disorders were higher among khat chewers, where 127 (71.8%) had symptoms of gastritis and 64 (36.2%) of them had dental problems, 86 (48.6%) had constipation, and 26 (14.7%) hemorrhoids, which demonstrated a significant association between khat chewing and gastrointestinal disorders [27]. 

Heavy and prolonged consumption of khat can also be associated with intestinal obstruction. A retrospective case series by Safadi MF was done between July 1, 2013, and December 31, 2015, in Yemen. These case series included all patients presented to a local hospital's emergency department in Yemen with intestinal obstruction symptoms. The study included seven patients (five males and two females) with a mean age of 41.4 years (22-60 years). All patients presented after 8-12 hours of prolonged khat chewing with abdominal pain, inability to pass stool, and severe abdominal distension. Laboratory results were normal apart from slight leukocytosis (<15 × 109/L) in three patients. The erect abdominal X-ray showed air-fluid levels. The management included intravenous fluids and symptomatic therapy; the symptoms resolved in 1-2 days, and the follow-up after one week showed no residual complaints [28]. 

Hepatitis*: *Khat components also have adverse effects on the hepatobiliary system. Based on two case reports by Jenkins MG et al., chewing khat regularly can induce liver tissue inflammation. The first case presents a 49-year-old Somali male with a four-week history of non-specific abdominal pain and abnormal liver function tests. The patient had no significant past medical or family history, and there was no history of alcohol use, no regular or over-the-counter medications, or use of other alternative remedies; also, he did not report any illicit drug. His blood work showed high alanine transaminase (ALT) 893 IU/l, bilirubin 100 μmol/l, alkaline phosphatase (ALP) 159 IU/l, and gamma-glutamyl transpeptidase (GGT) 212 IU/l, albumin 38 g/l, and international normalized ratio (INR) 1.0. He was negative for viral hepatitis (A, B, C, and E) and anti-nuclear, anti-smooth muscle, gastric parietal cell, mitochondrial, and liver/kidney microsomal autoantibodies. Serum ceruloplasmin, ferritin, and α-1-antitrypsin levels were all normal. Liver ultrasound was unremarkable. Further detailed history revealed that he had been chewing an unknown amount of khat regularly for several years for recreational purposes. A liver biopsy showed severe acute hepatitis, thought to be secondary to khat consumption as no other etiological factors could be identified. He was advised to abstain from chewing khat, and his liver function tests (LFTs) significantly improved within 7-10 days of abstinence, and he remained well for the next three years. Three years later, he re-presented to his general practitioner with a two-week history of similar complaints. The blood works showed the same picture of high liver enzymes and negative for viral markers. All protests began after the restarting of khat chewing. He was again advised to abstain from khat, and his LFTs improved gradually over eight weeks, returning to normal in six months [29]. 

The second case presented by Jenkins MG et al. has a similar presentation to the first one. A 39-year-old Somali male presented to the emergency department with a seven-day history of jaundice and dark urine, preceded by three weeks of intermittent right upper quadrant pain. He was previously well with no significant past medical and family history, and there was no history of alcohol use, no regular or over-the-counter medications or use of other alternative remedies, and he did not report any illicit drug use; however, he worked nights as a taxi driver and had used khat daily, over two hours at a time, for 3-4 years as a stimulant. Blood work was normal apart from high liver enzymes, but the liver biopsy showed peri-portal collapse, lobular and portal inflammation, and some features of interface hepatitis with eosinophils. However, his serum eosinophils count was normal. His symptoms resolved, and liver function tests improved over 7-10 days following abstinence from khat use, and a final diagnosis of acute hepatitis secondary to khat use was made. He re-presented five months later with another acute hepatitis following an episode of khat chewing. High liver enzymes were noted and adverse viral profile; a liver magnetic resonance imaging (MRI) was done, which showed further reduction in the size of the left lobe of the liver with new fibrosis in the right lobe [29] 

Liver cirrhosis*:* A case series conducted by Mahamoud HD et al. suggests that khat can cause liver cirrhosis in people who consume it regularly. Eight Somali men aged 27-70 years living in Somaliland were identified with an otherwise unknown cause. All chewed khat habitually for many years (15-128 bundles per day, years of use). A liver biopsy was consistent with khat hepatotoxicity, changes of chronic hepatitis, lobular cholestasis, and advanced fibrosis [30]. 

Type 2 diabetes and glycemia: An analytical, cross-sectional study was done by Badedi M et al. that included 472 Saudi participants randomly selected from primary healthcare centers in Jazan, Saudi Arabia]. The result showed that khat chewing prevalence was 29.3% in patients with type 2 diabetes mellitus (T2DM) in the studied population. Most of the khat chewers had similar doses and duration of khat chewing. A significant association was found between khat chewing and T2DM. In their study, the prevalence of poor glycemic control in patients with T2DM was 71%. Some studies show that khat influences blood sugar by destroying pancreatic beta cells; however, other studies found that khat chewing increased blood glucose levels in patients with T2DM [31]. More research is needed in this direction. 

Khat effects on appetite:* *As per Murray CD et al., khat significantly decreased subjective feelings of hunger and increased fullness (p<0.05) but did not affect ghrelin and peptide YY (PYY) levels. The anorexigenic effect of khat may be secondary to central mechanisms mediated via cathinone; however, further studies are necessary on the mechanism of action of khat's components and its anorexigenic effects [32]. 

Genito-Urinary and Reproductive System 

The effects of khat on kidneys and urine bladder: Mworia CM et al. investigated the possible effects of khat on the levels of various biochemical parameters to assess kidney and liver function. A cross-sectional study was done in Kenia; 391 people were enrolled in the study (198 khat chewers and 193 non-chewers). The result showed that chewing khat could damage the liver as total bilirubin, direct bilirubin, and alkaline phosphatase activity was significantly increased in the serum of khat consumers than non-consumers. While total creatinine was decreased, serum electrolytes were not affected, showing that khat chewing has no predisposing effect on renal disorders [33]. However, Shabbir A et al. investigated the impact of khat consumption on the liver and renal functions of the users of the Jazan region of Saudi Arabia. The study was mainly conducted to examine the people's liver and kidney functions using khat as a daily habit; blood was collected from 50 khat-users and 50 non-users. Khat-users had increased serum concentration of aspartate aminotransferase (AST), alanine aminotransferase (ALT), and alkaline phosphatase (ALP) as compared to the non-users, control. Additionally, serum creatinine concentration, urea, and uric acid were significantly higher in khat users than healthy non-users. Total fats and triglycerides levels were also relatively high in khat consuming people. Simultaneously, high-density lipoprotein (HDL) and low-density lipoprotein (LDL) values were low compared to the control group of non-users. This study showed that components present in khat are responsible for hepatic and renal toxicity [34]. 

Based on the study done by Nasher AA et al., khat can also derange the urodynamics through selective alpha 1-adrenergic receptors located in the neck of the urine bladder. The urodynamics of 11 healthy males was studied before and during a khat chewing preceded by indoramin hydrochloride or placebo. The result demonstrated that khat chewing produced a fall in average and maximum urine flow rate inhibited by indoramin [35]. 

The effects of khat on the reproductive system:* *Some studies on animals show that regular and heavy consumption of khat affects male fertility. Nyachieo A et al. investigated the effects of oral administration of high-dose khat on sperm parameters and male hormonal levels in olive baboons. Six male baboons received a high dose of khat (500 g/week) during a month, and sperm analysis along with plasma collection for hormonal analysis (testosterone, prolactin, and cortisol) was done weekly during one month of consumption and as well as two weeks after the last dose of khat. The results suggested that khat's high-dose decreases sperm quality and testosterone level and may contribute to male infertility [36]. 

Hakim LY evaluated khat's influence, alone and in combination with other drugs, on the qualitative characteristics of seminal fluid analysis of the male partners of allegedly infertile Ethiopian couples. A total of 214 male patients with a history of infertility and substance use of khat alone or in combination with tobacco smoking, coffee drinking, and alcohol intake of over one year. The study revealed decreased volume, sperm count, motility, and morphological changes in khat chewers compared to non-drug users, although the differences were not statistically significant (p>0.05) [37]. However, detailed studies on precise mechanisms by which khat may affect the male reproductive physiology have not been elucidated. 

*The Effects of Khat on Pregnant Woman and Fetus * 

Abdel-Aleem M. et al. enrolled in a prospective study 60 regular khat chewing pregnant women and 120 non-khat chewing pregnant women in Taiz, Republic of Yemen. Khat chewer pregnant women were having a statistically significant risk of six times for preterm labor, 3.83 times for labor induction and a statistically insignificant chance of 4.10 times for preeclampsia, 2.78 for blood transfusion and fetal distress, 2.05 for premature rupture of membranes (PROM), 2.03 for postpartum hemorrhage​​​​​​​ (PPH)** **and perineal tears and 2.02 for intrauterine fetal demise (IUFD). They had significantly lower mean hemoglobin concentration at delivery when compared with the control, statistically significant risk of 6.56 times for breech presentation; 8.94 times to deliver fetuses with low birth weight (<2500 gm); 6.0 times for neonatal admission to the intensive care unit (ICU), and significant risk of 3.54 times for perinatal mortality and 2.02 times higher risk for congenital malformations [38]. Further studies are required on khat using during pregnancy and its effects on mother and baby.

A pilot study by Kristiansson B et al. in 1987 found that nor-pseudoephedrine, one of khat's active ingredients, is excreted in breast milk and traced in one's urine breastfed infant [39]. Therefore, the use of khat during lactation should be discouraged until further studies will elucidate the effects of Khat on breastfeeding babies. 

Khat and Cancer 

The systematic review done by El-Zaemey S et al. in 2015 found that exposure to khat may be associated with potentially malignant and malignant oral disorders; however, methodological issues, such as inadequate study design, selection of study subjects, clinical evaluations of the outcome, and limited adjustment for confounders, sample size limits the strength of the evidence base in this area [40]. 

A pilot case-control study in Ethiopia by Leon ME et al. shows that khat use can be associated with esophageal cancer; however, further studies should be done to confirm this as increases in esophageal cancer were observed with ever tobacco use, alcohol consumption, low consumption of green vegetables, and salty diet [41]. 

Limitations of this review

This review article is a traditional review and, therefore, does not follow the standard Preferred Reporting Items for Systematic Reviews and Meta-Analyses (PRISMA) guidelines for systematic reviews. 

## Conclusions

The practice of chewing khat leaves (Catha edulis) is widespread in some regions of East Africa and the Arabian Peninsula. There is disquiet about health hazards related to the consumption of khat. Several studies have shown that khat contains chemicals such as cathinone and cathine, which are active brain stimulants. It has pleasing central stimulant properties, commonly believed to improve work capacity and prevent fatigue. 

According to the studies done over the past years, khat harms the human body. There is an apparent adverse effect on the cardiovascular system, central nervous system, pregnant women, and oropharynx. However, more research is essential on the direction of heavy khat consumption and its effect on cardiac rhythm, on the pancreas and glycemic level, understanding the mechanism of action involved in its anorexigenic development, and the effect involved in renal toxicity. Despite studies done on khat's results on the reproductive system, there is still a need for detailed reviews of precise mechanisms by which khat may affect the male reproductive physiology. Additionally, more research is needed on obstetric complications, teratogenicity, and infant death. To confirm that khat alone is a harmful plant, scientists must do case-control studies. 

Studies lack khat's effects on breastfed babies, and there is no knowledge about khat's reaction to the female reproductive system (ovaries and hormonal profile). There is no scientific information about khat interaction with drugs, and there is still no information about the safe dosage of khat consumption. 

Health professionals should educate users about potential harms arising from khat use, promoting the drug's responsible use to minimize the individual and the community's adverse health effects. Further training of health practitioners, including drug and alcohol service practitioners, is necessary to improve khat's awareness and health consequences. 
